# Three New Phases of Repairing DNA Damage in E. coli

**DOI:** 10.1371/journal.pbio.0030239

**Published:** 2005-06-21

Any cell that receives a dose of radiation is placed in a dangerous situation. The DNA damage resulting from exposure to such radiation (or any other mutagen) can cause massive rearrangements to genetic information and potentially kill the cell. Bacteria have learned to cope with this threat by activating genes that repair DNA damage and by preventing a cell from dividing before these repairs are completed. In the bacteria Escherichia coli, these repair genes form what is known as the SOS response.

The E. coli SOS response has been used to study DNA repair for decades, and a great deal is known about how the more than 30 genes involved in the response function. Two proteins figure prominently in this response. The LexA protein acts as a repressor and inhibits the expression of SOS genes under normal conditions; in the event of DNA damage, the protein RecA inactivates the LexA repressor by enhancing its autocleavage into two fragments, which initiates the SOS response. While these initial stages are well understood, how all the SOS genes are coordinated, and ultimately turned off, is only beginning to be explored.

In a new study, Joel Stavans, Uri Alon, and colleagues have closely followed the SOS response in individual E. coli cells to investigate its dynamics. Previous studies, which monitored the temporal pattern of activation of entire populations of cells, found that SOS genes turned on in one peak upon DNA damage. But Friedman et al. found that SOS genes in individual bacteria respond to DNA damage in three precisely timed phases. This observation reveals the importance of examining complex processes at the level of single cells, while furthering our understanding of how the SOS response is structured in E. coli. [Fig pbio-0030239-g001]

**Figure pbio-0030239-g001:**
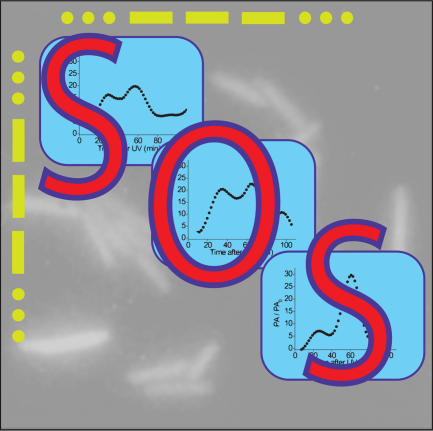
Genes involved in the SOS response to DNA damage are expressed in three precisely timed phases

Friedman et al. monitored the SOS response by attaching a green fluorescent protein (GFP) to the promoters (the section of DNA responsible for activating a gene) of three SOS genes *(lexA, recA,* and *umuDC)*. Bacteria expressing these promoter-GFP fusions became fluorescent within minutes of being exposed to UV radiation, visualized using time-lapse fluorescence microscopy. Since GFP fluorescence is directly correlated with the expression of each of the chosen genes (i.e., their promoter activity), the authors could gauge the SOS response rate upon DNA damage.

To induce the SOS response, the authors exposed E. coli cells to UV radiation. By monitoring individual cells at two-minute intervals after this dose, Friedman et al. found up to three peaks of promoter activity at roughly 30, 60, and 100 minutes. Although the amount of this activity and the average number of peaks varied between cells, the timing was always similar in different cells, suggesting a highly structured, timed response. When the authors averaged this response over the population, it “washed out” into a single peak—which explains why the three peaks of expression were not previously detected.

A deeper look into the dynamics of the SOS response in single E. coli cells showed that it did not correlate with cell size, suggesting the SOS response is not synchronized with the cell cycle. In addition, Friedman et al. repeated their experiments in a bacterial strain lacking the SOS response gene *umuDC*. The peak pattern was altered in this mutant strain, and the precision in the appearance of the peaks was reduced. By re-examining the SOS response in single cells, Friedman et al. have visualized an accurately timed and synchronized DNA repair process. Modulations in response to DNA damage have also been observed recently in individual mammalian cells. Future experiments in E. coli—one of the most genetically tractable model systems—should help explain how this timed response is related to the different pathways of DNA repair and shutoff of the response.

